# Effects of genus *Epimedium* in the treatment of osteoarthritis and relevant signaling pathways

**DOI:** 10.1186/s13020-023-00788-8

**Published:** 2023-07-31

**Authors:** Xue Tong, Yan Wang, Benchao Dong, Yan Li, Shuang Lang, Jianxiong Ma, Xinlong Ma

**Affiliations:** 1 Orthopaedics Institute of Tianjin, Tianjin Hospital, Tianjin, China; 2 School of Integrative Medicine, Tianjin University of Traditional Chinese Medicine, Tianjin, China; 3 Tianjin Hospital, Tianjin University, Tianjin, China

**Keywords:** Osteoarthritis, Traditional Chinese Medicine, *Epimedium*, Ingredient, Effects, Signaling pathway

## Abstract

Osteoarthritis (OA) is a common chronic degenerative joint disease in clinical practice with a high prevalence, especially in the elderly. Traditional Chinese Medicine (TCM) believes that OA belongs to the category of “Bi syndrome” and the “bone Bi syndrome”. The etiology and pathogenesis lie in the deficiency of the liver and kidney, the deficiency of Qi and blood, and external exposure to wind, cold, and dampness. *Epimedium* is a yang-reinforcing herb in TCM, which can tonify the liver and kidney, strengthen muscles and bones, dispel wind, cold and dampness, and can treat both the symptoms and the root cause of “bone Bi syndrome”. In addition, *Epimedium* contains a large number of ingredients. Through modern science and technology, more than 270 compounds have been found in *Epimedium*, among which flavonoids are the main active ingredients. Therefore, our study will review the effects and mechanisms of genus *Epimedium* in treating OA from two aspects: (1) Introduction of *Epimedium* and its main active ingredients; (2) Effects of *Epimedium* and its active ingredients in treating OA and relevant signaling pathways, in order to provide more ideas for OA treatment.

## Background

Osteoarthritis (OA) is a chronic degenerative disease with a significantly increased prevalence with age. OA can lead to disability, severely affecting patients’ life quality. Results from a Global Burden of Disease 2010 Study as early as 2010 showed that hip and knee OA ranked 11th out of 291 diseases causing disability and 38th out of disability-adjusted life years [1]. During 2013–2015, 54.4 million adults in the United States had arthritis, of those, 23.7 million (43.5%) were limited in their daily activities due to arthritis [2]. Besides, the high prevalence and disability rates have added to the burden on families and the entire healthcare system. According to statistics, the annual medical cost of knee OA exceeds $27 billion, and total knee arthroplasties-related expenditures have exceeded $11 billion annually [3]. China is one of the world’s most populous countries and is in the stage of rapid aging. Many scholars believe that OA will become one of the major problems of the Chinese health system [4]. Therefore, there is an urgent need for more research to be devoted to OA pathology and treatment.

Currently, the treatment methods of OA can be divided into non-drug intervention, drug treatment and surgical treatment [5]. Non-drug intervention mainly focuses on education, exercise and weight loss. Drug treatment includes the use of anti-inflammatory drugs, such as oral or topical non-steroidal anti-inflammatory drugs and cyclooxygenase-2 inhibitors, intra-articular injections of corticosteroids or hyaluronic acid, and the use of pain relievers such as duloxetine or opioids. Surgical treatment is the ultimate option for patients with advanced OA or persistent pain [5]. However, the benefit/risk ratio of these commonly used methods may not be high [6]. In Kloppenburg’s study, he listed a number of studies on current drugs commonly used in the clinic to treat OA [6]. These clinical results indicate that their effects on pain and functional improvements in OA patients are relatively small, especially in terms of long-term effects and pain relief, and intra-articular injection with corticosteroids may lead to cartilage loss. In addition, some drugs have an increased risk of adverse events such as gastrointestinal ulcers, bleeding, hyperglycemia, and cardiovascular disease [5, 6].

After thousands of years of development and inheritance, Traditional Chinese Medicine (TCM) has formed a medical system with unique theories and methods. During the COVID-19 epidemic, TCM has played an essential role in fighting against the virus [7, 8]. In the treatment of OA, TCM also has many insights and great potential.

**Fig. 1:**
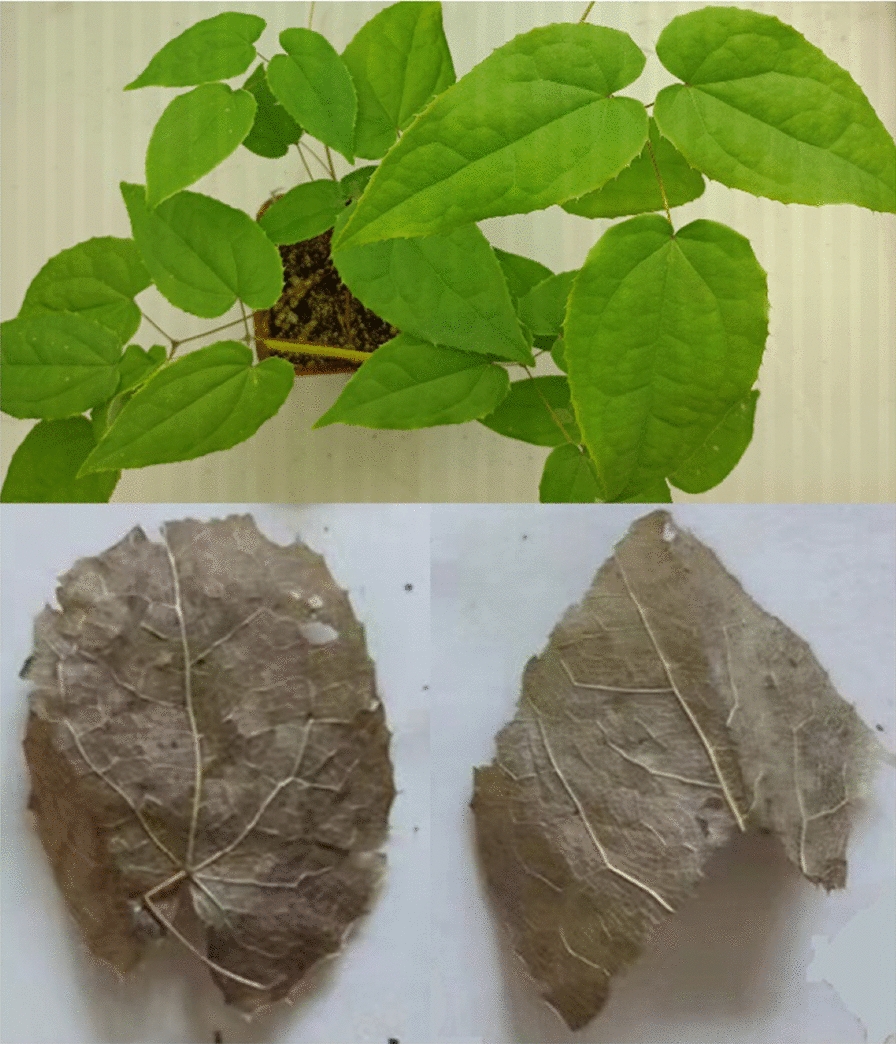
*Epimedium* plant and processed *Epimedium*

OA is classified as “Bi syndrome” and “bone Bi syndrome” in TCM [9]. The primary pathogenesis is the deficiency of Qi, blood, Ying, and Wei, and external exposure to wind, cold and dampness, resulting in the stagnation of Qi, blood and meridians, and then causing pain [9]. *Epimedium*, as shown in Fig. 1, is a Yang tonic herb in TCM, and the central medicinal part is dried leaves. Its effects are good at tonifying the kidney and Yang, strengthening the muscles and bones, and dispelling wind and dampness, which means that *Epimedium* can treat both the symptoms and the root cause of “bone Bi syndrome”. Therefore, our article will review *Epimedium* and its main active ingredients, and their roles and mechanisms in OA treatment, in order to provide more ideas for OA study.

## *Epimedium* and its main active ingredients

The genus *Epimedium* (Yinyanghuo in Chinese) belongs to the family *Berberidaceae* Juss., and was initially documented in “Shen Nong Ben Cao Jing” [10]. At present, as many as 80 species of *Epimedium* have been discovered, most of which are endemic to China and mainly distributed in the southwest and central regions [11, 12]. There are also a few species in eastern, southern and central Asia and Europe. In the Chinese Pharmacopoeia (2020 edition), *Epimedium brevicomu* Maxim., *Epimedium sagittatum* (Sieb. et Zucc.) Maxim., *Epimedium pubescens* Maxim., and *Epimedium koreanum* Nakai were considered as the main medicinal species [13]. In fact, besides these species, species such as *Epimedium wushanense* T.S.Ying and *Epimedium acuminatum* Franch. are also widely used [14]. Due to the good medicinal properties of *Epimedium*, many proprietary Chinese medicines and health products that have been marketed contain *Epimedium*, of which *Epimedium* as the main medicine are shown in Table 1. They are used for the treatment of osteoporosis, reproductive system disorders, kidney yang deficiency symptoms, and other diseases. With the increasing research on *Epimedium* and its ingredients, medical scientists have also found that they have the potential to treat uveitis [15], cardiovascular disease [16], tumors [17, 18], influenza A [19], and neuroprotection [20, 21] through anti-inflammation [22], antioxidant [20], regulating cell proliferation, apoptosis and autophagy [16, 23], modulating immunity [24], and antiviral [19] mechanisms.

**Table 1:** Chinese patent medicine with *Epimedium* as the main medicinal material

Name	Components	Indication	Drug safety	National drug standard (standard number)
XianLing Gubao capsule	*Epimedium*, Xu-duan* and other 4 Chinese medicines	Osteoporosis, fractures, OA, aseptic bone necrosis	Receptor X	WS-10269(ZD-0269)-2002-2011Z
Gusongbao capsule	*Epimedium*, Chi-shao* and other 7 Chinese medicines	Fractures and bone pains caused by osteoporosis	Receptor X	YBZ01612003-2006
*Epimedium* total flavonoids capsule	*Epimedium* total flavonoids extraction	Primary osteoporosis with kidney-yang deficiency syndrome	Receptor X	YBZ00132013
Yanghuo Sanqi capsule	*Epimedium*, San-qi*	Thoracic obstruction caused by yang deficiency and blood stasis	Receptor X	YBZ01792009
Yiqi Bushen capsule	Dang-shen*, *Epimedium* and other 7 Chinese medicines	Neuroasthenia or climacteric syndrome with spleen and kidney deficiency syndrome	Receptor X	WS-11413(ZD-1413)-2002
Xianlingpi tablet/capsule	*Epimedium*	Impotence and spermatorrhea, flaccidity of muscule and bone, rheumatic arthralgia	Receptor X	WS-10707(ZD-0707)-2002-2011Z/YBZ23752005-2009Z
Tenghuang Jiangu Pill	Shu-di-huang*, Lu-xian-cao*, Gu-sui-bu*, Rou-cong-rong*, *Epimedium* and other 2 Chinese medicines	Hypertrophic spondylitis, cervical spondylosis, heel spur, proliferative arthritis, Kashin-Beck disease	Receptor X	WS-11061(ZD-1061)-2002-2012Z
Zhuanggu Guanjie capsule	Shu-di-huang*, *Epimedium* and other 10 Chinese medicines	Degenerative OA and lumbar muscle strain caused by liver and kidney deficiency, qi stagnation and blood stasis, and meridians obstruction	Receptor X	WS3-709(Z-141)-2010Z
Huoyang Bushen capsule	Ren-shen*, cartialgenous, *Epimedium* and other 13 Chinese medicines	Syndrome of kidney yang deficiency	Receptor X	WS-5739(B-0739)-2014Z
Hugu capsule	He-shou-wu*, *Epimedium* and other 8 Chinese medicines	Primary osteoporosis	Receptor X	YBZ12742004-2009Z
Compound *Epimedium* oral liquid	*Epimedium*, Shu-di-huang*, and other 6 Chinese medicines	Syndrome of kidney yang deficiency	Over the counter	WS-5357(B-0357) -2002
Huolong Bushen oral liquid	*Epimedium*, Hai-long* and other 11 Chinese medicines	Syndrome of kidney yang deficiency	Over the counter	WS-5668(B-0668)-2002
Bushen Qiangshen capsule	*Epimedium*, Jin-ying-zi* and other 3 Chinese medicines	Kidney deficiency syndrome	Over the counter	WS3-B-0751–91
Yinyangsuo capsule/granule	*Epimedium*, Wu-wei-zi*	Impotence and premature ejaculation in male disease	Receptor X	YBZ01852008/YBZ24472005

*Are the pin-yin names of the medicinal materials, and their corresponding Latin names are as follows: Xu-duan, *Dipsacus asper* Wall. ex DC.; Chi-shao, *Paeonia anomala subsp. veitchii* (Lynch) D.Y.Hong & K.Y.Pan; San-qi, *Panax notoginseng* (Burkill) F.H.Chen ex C.Y.Wu & K.M.Feng; Dang-shen, *Codonopsis pilosula* (Franch.) Nannf. or *Codonopsis pilosula subsp. Pilosula* or *Codonopsis pilosula subsp. tangshen* (Oliv.) D.Y.Hong; Shu-di-huang, *Rehmannia glutinosa* (Gaertn.) DC.; Lu-xian-cao, *Pyrola calliantha* Andres or *Pyrola decorata* Andres; Gu-sui-bu, *Drynaria roosii* Nakaike; Rou-cong-rong, *Cistanche deserticola* Ma or *Cistanche phelypaea* (L.) Cout.; Ren-shen, *Panax ginseng* C.A.Mey.; He-shou-wu, *Reynoutria multiflora* (Thunb.) Moldenke; Hai-long, *Solenognathus hardwicki* (Gray) or *Syngnathoides biaculeatus* (Bloth) or *Syngnathus acus* Linnaeus; Jin-ying-zi, *Rosa laevigata* Michx.; Wu-wei-zi, *Schisandra chinensis* (Turcz.) Baill. or *Schisandra sphenanthera* Rehder & E.H.Wilson

**Fig. 2:**
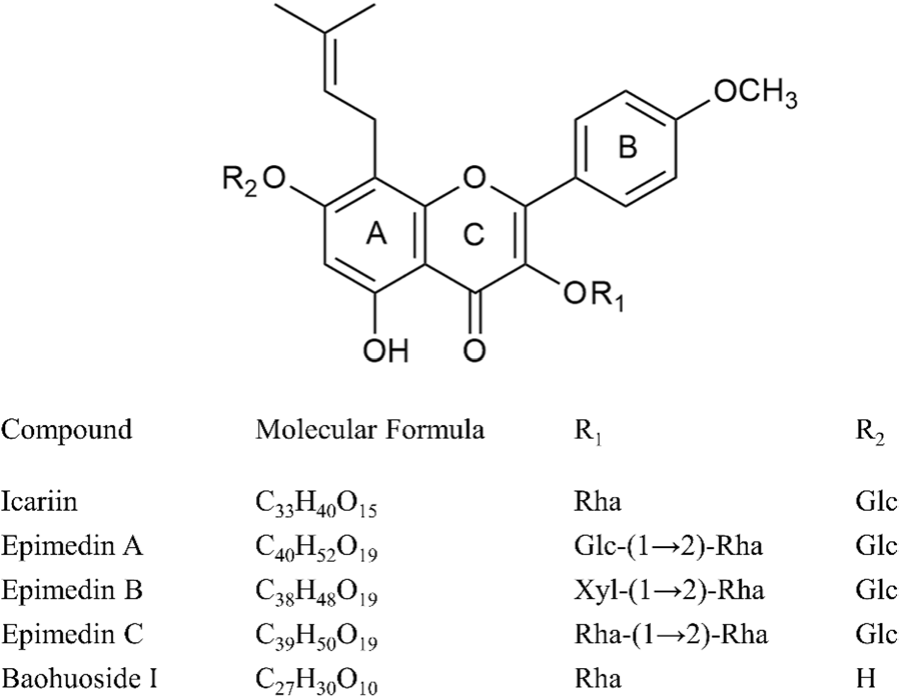
Skeleton of Flavonoids and the five main ingredients in *Epimedium*. “Glc” refers to glucose, “Rha” refers to rhamnose, and “Xyl” refers to xylose

*Epimedium* contains a large number of ingredients. According to statistics, more than 270 ingredients have been identified from different species of *Epimedium*, including flavonoids, lignans, polysaccharides, alkaloids, and volatile oils [14, 25]. Among them, flavonoids account for more than half of the ingredients, which shows that flavonoids are the vital material basis for *Epimedium* to function [12]. Flavonoids refer to a series of compounds formed by the interconnection of two benzene rings through three carbon atoms, that is, the C6–C3–C6 structure, as shown in Fig. 2 [26, 27]. In *Epimedium*, prenylated flavonols and their glycosides are the most distinctive compounds, and the basis of *Epimedium*’s efficacy, such as icariin, epimedin A, epimedin B, epimedin C, and baohuoside I [25, 28]. Icariin and epimedins A–C are also specified in the Chinese Pharmacopoeia as quality control standards for crude and processed products of *Epimedium* [13]. The structures of these five main ingredients in *Epimedium* are shown in Fig. 2. However, the absorption permeability of these compounds is not high, so their bioavailability is actually very low [29, 30]. A higher number of glycosyl groups means higher solubility, but poorer permeability. Baohuoside I (monoglycoside), icariin (diglycoside) and epimedins A–C (triglycosides) have sequentially lower permeability [29, 31]. In different research models and different body fluids, these compounds undergo complex chemical transformations, and deglycosylation is a crucial process for metabolism and absorption in vivo [25, 30]. Epimedins A–C can convert to secondary glycosides and eventually to monoglycosides or aglycone. Icariin can deglycosylate to monoglycosides or aglycone, and baohuoside I can convert to aglycone [25, 31]. Baohuoside I and icaritin (aglycone) are the main metabolites of icariin, of which, icaritin is not soluble enough in water due to the absence of sugar substitution, which greatly limits its research and application [28, 29, 32]. Therefore, improving the bioavailability of these flavonoids is a prerequisite for better performance of their effects. Szabo et al. summarized some of the current methods to improve the bioavailability of icariin and its derivatives by searching a large number of literature [33]. These methods include phospholipid complex formation, micelle formation, etc. They also believed that the simultaneous application of multiple methods may increase the bioavailability of sparingly soluble flavonoids exponentially [33]. In the Processing of Chinese Materia Medica, the most commonly used processing method of *Epimedium* is to stir-fry it with suet oil [34]. Studies have shown that stir-frying with suet oil can transform polyglycosides to secondary glycosides in *Epimedium*, which is more conducive to absorption [29, 35]. Moreover, the addition of suet oil can further improve the bioavailability of *Epimedium* flavonoids through the formation of self-assembled micelles [30].

Besides flavonoids, lignans, polysaccharides and alkaloids are also important ingredients in *Epimedium*. These ingredients have been shown to have anti-inflammatory [36], antioxidant [37, 38], and immunomodulatory activities [39]. In addition, *Epimedium* polysaccharides have also been shown that can increase the solubility of icariin and baohuoside I in a concentration-dependent manner, and the solubilizing effect of acidic polysaccharides was more pronounced [40]. Magnoflorine is the main alkaloid contained in *Epimedium* [41]. Gao et al. determined the content of alkaloids in the leaves of 29 species of genus *Epimedium*, and found that the content of magnoflorine ranged from 0.003 to 2. 603%, with the highest content value being 867 times higher than the lowest content value [42]. *Epimedium* species with similar magnoflorine contents often have consanguinity or belong to the same taxon. Therefore, they believed that the content of magnoflorine can be used as an evidence to identify the subgenus relationship of *Epimedium* [42].

However, there are few studies of these ingredients in the treatment of OA. Through searching and summarizing a large number of studies, we found that the researches on *Epimedium* still focus on flavonoids compounds, especially in treating OA. Therefore, in the following sections, we will introduce in detail the effects and mechanisms of *Epimedium* and its ingredients, mainly flavonoids, in the OA treatment. Meanwhile, based on the anti-inflammatory and antioxidant activities of lignans and polysaccharides from *Epimedium*, and there having been studies on the treatment of OA with these components from other herbs, such as lignans of *Schisandra chinensis* (Turcz.) Baill. [43], total lignans of *Vitex negundo var. cannabifolia* (Sieb.et Zucc.) Hand.-Mazz. seeds [44], polysaccharides of *Achyranthes bidentata* Blume [45], and polysaccharides of *Eucommia ulmoides* Oliv. [46], we expect more *Epimedium* ingredients can participate in the research of OA and other diseases in the future.

## Study on the mechanisms of *Epimedium* on OA

OA is the most common type of arthritis. All joint tissues can involve in the pathogenesis and progression of OA, including cartilage, synovium, subchondral bone, joint capsule and ligaments. The degeneration of cartilage and the loss of its biomechanical properties are the core pathological changes and outcomes of OA [47, 48]. Therefore, our study will summarize the recent in vivo and in vitro experimental studies in treating OA by *Epimedium* and its active ingredients from the perspective of cartilage, synovium and subchondral bone.

### Maintaining structures and functions of articular cartilage

Normal articular cartilage consists of a small number of chondrocytes and a large amount of extracellular matrix [49]. The extracellular matrix is mainly composed of type II collagen (Col2) and proteoglycans [49]. Other collagens such as IX and XI, also play a vital role in cartilage development and function [50]. Proteoglycan is a complex sugar composed of protein and glycosaminoglycan (GAG), which plays a protective role on the skeleton formed by collagen [51].

Chondrocytes are dispersed in the matrix and maintain the synthesis and catabolism of extracellular matrix. The degradation of extracellular matrix mainly depends on the production of a series of proteases by chondrocytes [52]. Matrix metalloproteinases (MMPs) and aggrecanases are the key enzymes that perform the degradation and are the major mediators of OA matrix loss [49]. Among them, MMP13 is a collagenase with the strongest effect, which can directly degrade Col2 and cause matrix loss [53]. Aggrecan-degrading enzymes are represented by ADAMTS (ADAM with thrombospondin-1 domains)-4 and ADAMTS-5 [49].

**Fig. 3:**
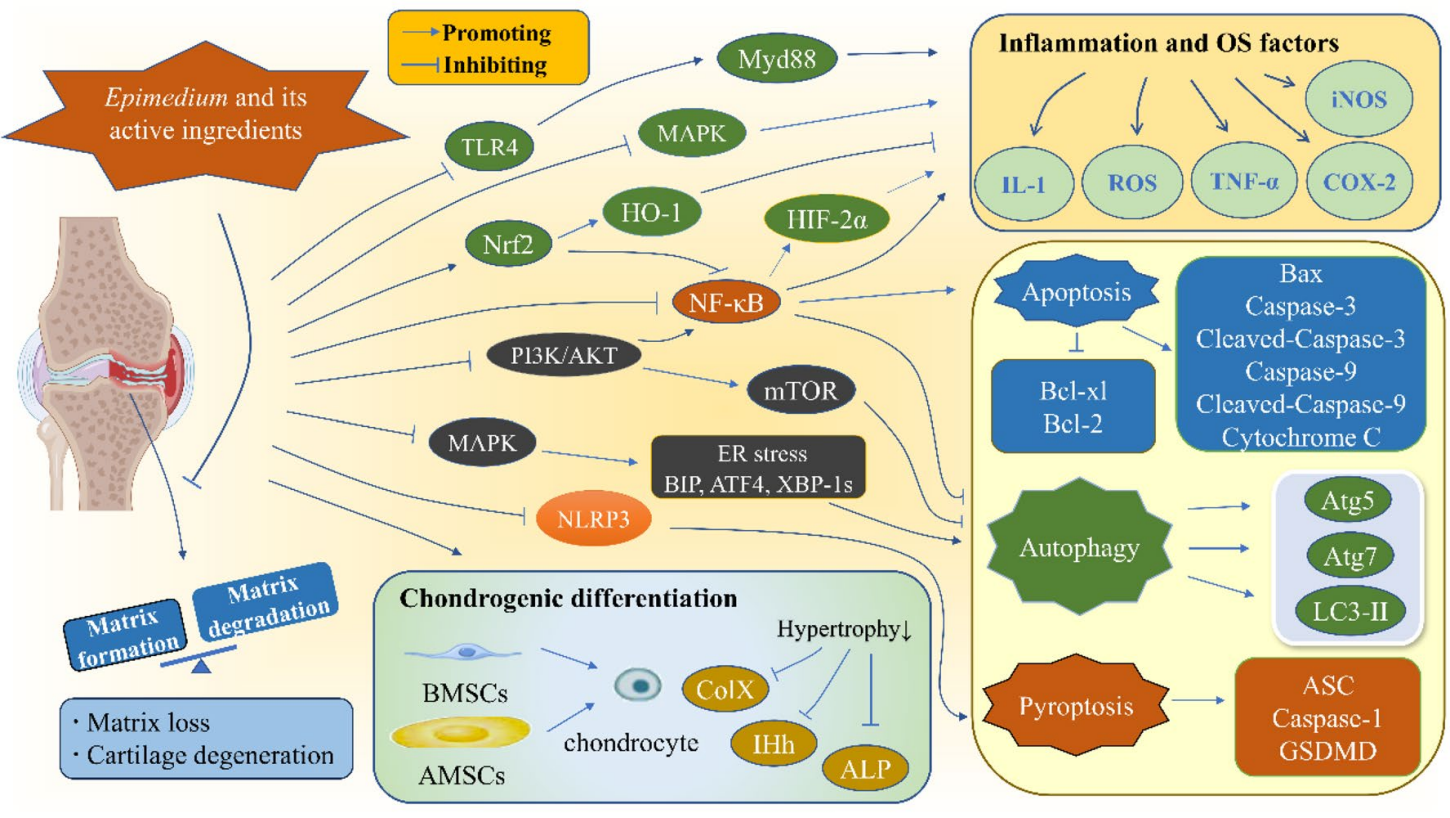
Effects and relevant signaling pathways of *Epimedium* and its active ingredients in maintaining the structures and functions of cartilage to treat OA

Chondrocytes also control the matrix formation. Aggrecan is the most abundant proteoglycan in the matrix synthesized by chondrocytes [52]. It can interact with hyaluronic acid to provide osmotic properties for cartilage, and is considered as one of the markers of matrix formation [52]. However, the turnover rate of chondrocytes and matrix is extremely low, so articular cartilage has been considered as an inert tissue for a long time [54]. Chondrocytes are the only resident cell group in articular cartilage [54, 55]. They lack the nourishment of nerves, blood vessels and lymph, and are separated from each other by matrix, so they are vividly called solitary cells [54, 55]. Besides, they are highly differentiated cells, which means that once damaged, the self-healing ability of cartilage tissue is very poor [51]. Studies have shown that many pathological events occur in articular cartilage during the progression of OA, including inflammation, oxidative stress (OS), increased apoptosis, and decreased autophagy [56]. Eventually, the matrix is lost and the cartilage degenerates. Table 2 summarizes the literature about *Epimedium* and its active ingredients in treating OA from the aspect of articular cartilage. It can be seen that they can maintain structures and functions of cartilage from the following aspects: (1) anti-inflammation and anti-OS; (2) inhibiting chondrocyte apoptosis and pyroptosis, and improving autophagy; (3) promoting chondrogenic differentiation of mesenchymal stem cells (MSCs), as shown in Fig. 3.

**Table 2:** Studies of *Epimedium* and its active ingredients in maintaining the structures and functions of cartilage to treat OA

References	Assay type	Component of *Epimedium*	Concentration/dosage	Cells/model	Effects	Mechanisms
Anti-inflammation and anti-OS						
Ziadlou [53]	In vitro	Epimedin C	25 µM	Human knee OA chondrocytes treated by IL-1β/TNF-α	Matrix degradation: MMP1↓, MMP3↓, MMP13↓ Inflammatory factors: COX-2↓ Matrix formation: Col2↑, aggrecan↑, GAG/DNA↑	Promoting the extracellular matrix formation through anti-inflammatory
Wang [57]	In vitro	Icariin	1 µM	Mouse chondrocytes (ADTC5) treated by TNF-α	Chondrocyte viability↑ Signal pathway: p-IKKα/β/IKKα/β↓, p-IκB/IκB↓, p-NF-κB/NF-κB↓, HIF-2α↓	Relieving articular cartilage damage by inhibiting NF-κB/HIF-2α signaling pathway to reduce inflammation
	In vivo	3D alginate-gelfoam complexes incorporated with Icariin	1 µM	Knee cartilage defect mouse model	Histopathology: relatively intact cartilage surface, substantial subchondral bone, ICRS II score↑ Matrix formation: Sox9↑, aggrecan↑, Col2α↑ Matrix degradation: MMP9↓, ADAMTS-5↓ Signal pathway: HIF-2α positive cells↓, NF-κB positive cells↓	
Sun [58]	In vitro	Hyperoside	10, 20 and 40 μM	Il-1β stimulated C57BL/6 mouse chondrocytes	Inflammatory factors: iNOS↓, COX2↓ Matrix degradation: ADAMTS5↓, MMP3↓, MMP13↓ Matrix formation: Sox9↑, Col2↑, aggrecan↑ OS factor: ROS↓ Apoptosis-related proteins: Bcl-xl↑, Bax↓, Cytochrome C↓, cleaved-caspase-9↓, cleaved-caspase-3↓ Signal pathway: p-PI3K/PI3K↓, p-AKT/AKT↓, p-ERK/ERK↓, p-JNK/JNK↓, p-C-JUN↓, p-p65/p65↓, p-IκBα↓, IκBα↑, Nrf2↑, HO-1↑	Alleviating OA inflammation and matrix loss by inhibiting PI3K/AKT/NF-κB and MAPK signaling pathways, while enhancing Nrf2/HO-1 signaling pathway; anti-apoptosis of chondrocytes through Nrf2/ROS/Bax/Bcl-xl axis
	In vivo	Hyperoside	20 mg/kg	OA model established by the destabilized medial meniscus surgery in C57BL/6 mice	Histopathology: smoother and more intact cartilage surface, OARSI score↓ Signal pathway: Nrf2 positive cells↑	
Zuo [59]	In vitro	Icariin	10^–3^ µM	IL-1β stimulated human chondrocytes (icariin pretreatment)	Matrix formation: GAG↑ Matrix degradation: MMP3↓, MMP9↓, MMP13↓, ADAMTS-4↓ Anti-OS factors: Nrf2↑, SOD-1↑, SOD-2↑, GPX↑, NQO-1↑, HO-1↑, Keap1↓ OS factor: ROS↓	Inhibiting OA matrix degradation by Nrf2/ARE signaling pathway
Zeng [60]	In vitro	Icariin	20 μM	IL-1β-stimulated human SW1353 chondrosarcoma cells	Matrix degradation: MMP1↓, MMP3↓, MMP13↓ Signal pathway: p-p38↓, p-pERK↓, p-pJNK↓	Playing a role in cartilage protection by inhibiting the MAPK signaling pathway
Li [61]	In vivo	Icariin	20 mg/kg	Knee OA rat model established by the modified Hulth method	Histopathology: increase of cartilage thickness and bone trabeculae, smoother cartilage surface Micro-CT: BV/TV↑, BMD↑ Bone resorption markers: TRACP-5b↓, CTX-1↓ Bone formation markers: 1,25(OH)_2_D_3_↑, OCN↑ Bone remodeling related indicator: TGF-β↓, BMP-4↓, Smad4↓, p-Smad1/5/9/Smad1/5/9↓ Pain-related indices: TWL↑, MWT↑ Inflammatory factors: IL-1β↓, TNF-α↓ Matrix degradation: MMP3↓, MMP13↓	Treating OA and OA pain by the inhibition of TLR4/MyD88/NF-κB signaling pathway and neuromodulation
	In vitro	Icariin	20 μM	LPS-induced knee joint chondrocytes of SD rats	Matrix formation: Col2↑ Matrix degradation: MMP3↓ Signal pathway: TLR4↓, MyD88↓, TRAF-6↓, NF-κB p65↓, IKK-α↓, IKK-β↓	
	In vivo	Icariin	20 mg/kg	Knee OA rat model established by the modified Hulth method	Neuropeptides: NPY↓, NPY1R↓, SP↓, 5-HT1B R↓, VIP↑ Resting-state fMRI: decrease of ReHo in the cortex, hypothalamus and midbrain, increase of the hippocampus ReHo Pain-related genes: Inα↑, Rtn4↓	
Inhibiting chondrocyte apoptosis, pyroptosis, and improving autophagy						
Mi [62]	In vitro	Icariin	10 μM	TNF-α treated chondrocytes in SD rats	Inflammatory factors: IL-1↓, IL-6↓, IL-12↓ Autophagy markers: Atg 5↑, Atg 7↑, LC3-II↑ Delay cell cycle arrest Apoptosis-related proteins: Bcl-2↑, Bax↓, caspase-3↓, caspase-9↓ OS factors: NO↓, ROS↓ Matrix degradation: MMP 3↓, MMP 9↓ Signal pathway: p-p65/p65↓, IκBα↑	Anti-apoptosis and enhancing autophagy in chondrocytes by the inhibition of NF-κB signaling pathway
Wang [63]	In vitro	Icariin	30 μM	IL-1β stimulated human chondrocyte cell line CHON-001 and mouse chondrocyte cell line ATDC5	lncRNA: CYTOR↑ Cell apoptosis: apoptosis rate of chondrocytes↓ Inflammatory factors: IL-6↓, IL-8↓, TNF-α↓	Inhibiting chondrocyte apoptosis by the up-regulation of CYTOR
Zhou [64]	In vitro	*Epimedium* ethanol extraction	80 mg/L	SD rat chondrocytes of the knee joint	Histopathology: improvement of chondrocyte morphology Cell apoptosis: early apoptosis rate↓, late apoptosis and necrosis rate↓, total apoptosis rate↓	The inhibition of chondrocyte apoptosis
Zu [56]	In vitro	Icariin	5, 10 and 20 μM	LPS-treated Wistar rat chondrocytes of knee joint	Inflammatory factors: IL-1β↓, IL-18↓ Matrix degradation: MMP1↓, MMP13↓ Matrix formation: Col2↑ Pyroptosis-related proteins: NLRP3↓, ASC↓, caspase-1↓, GSDMD↓	Inhibiting chondrocyte pyroptosis mediated by NLRP3
	In vivo	Icariin	20 μM, 0.3 mL	Intra-articular injection of monosodium iodoacetate in Wistar rats	Histopathology: reduction of cartilage erosion Pyroptosis-related proteins: NLRP3 positive cells↓, NLRP3↓ Inflammatory factors: IL-1β↓, IL-18↓ Matrix degradation: MMP1↓, MMP13↓ Matrix formation: Col2↑	
Tang [65]	In vitro	Icariin	40, 60 and 80 μM	SD rat chondrocytes of the knee joint	Cell apoptosis: apoptosis rate of chondrocyte↓ Autophagy markers: ATG7↑, LC3-II/LC3-I↑ Signal pathway: PI3K↓, p-AKT1/AKT1↓, p-mTor/mTor↓, p-p70S6K/p70S6K↓	Enhancing autophagy and alleviating chondrocytes apoptosis through the inhibition of PI3K/AKT/mTOR signaling pathway
	In vivo	Icariin	20, 40 and 80 mg/kg	Knee OA rat model established by the modified Hulth method	Histopathology: OARSI score↓ Autophagy markers: Beclin-1↑, ATG7↑, LC3-II/LC3-I↑ Signal pathway: PI3K↓, p-AKT1/AKT1↓, p-mTor/mTor↓, p-p70S6K/p70S6K↓	
Chen [66]	In vitro	Icariin	40 μM	IL-1β-mediated human SW1353 cells (icariin pretreatment)	Matrix degradation: MMP3↓ Matrix formation: Col2↑ Autophagy markers: LC3-II/LC3-I↑, Beclin-1↑, p62↓, ULK1↑ Signal pathway: p-PI3K/PI3K↓, p-AKT/AKT↓, p-mTor/mTor↓	Alleviating OA by PI3K/Akt/mTOR/ULK1 signaling pathway
Liu [67]	In vitro	Icariin	0.1, 1 and 10 μM	Oxidation, glucose and serum deprivation-induced rabbit BMSCs (Icariin pretreatment)	ER stress-related proteins: BIP↓, ATF4↓, CHOP↓, p-IRE1a↓, XBP-1s↓ Apoptosis-related proteins: caspase-3↓, cleaved caspase-3↓, PARP↓, cleaved-PARP↓ Autophagy marker: LC3-I↑, p62↑, Beclin-1↓, ATG5↓, LC3 II↓ Signal pathway: p-ERK↓, p-P38↓, p-JNK↓	Inhibiting ER stress in BMSCs, thereby reducing apoptosis and autophagy through the inhibition of the MAPK signaling pathway
Wang [68]	In vitro	Icariin	1 µM	Alginate-C57BL/6 mouse chondrocytes 3D complex (Anoxic model)	Matrix formation: proteoglycan↑, Sox9↑ Chondrocyte viability↑ Signal pathway: HIF-1α↑ Glucose transport and anaerobic glycolytic enzymes: GLUT1↑, PGK1↑, PDK1↑, G6PD↑	Promoting HIF-1α expression and anaerobic glycolysis to increase chondrocyte viability
Promoting chondrogenic differentiation of mesenchymal stem cells						
Zhu [69]	In vitro	Icariin-loaded hydrogel	29.6, 147.8 and 738.9 μM	BMSCs	Cell proliferation and viability↑ Chondrogenic markers: Sox 9↑, Col2α1↑, aggrecan↑, HIF-1α↑ Signal pathway: Dvl1↑, β-catenin↑, GSK-3β↓	Promoting BMSCs chondrogenic differentiation through Wnt/β-catenin signaling pathway
	In vivo	Icariin-loaded hydrogel + BMSCs	147.8 μM, 0.4 mL	OA rat model established by destabilizing medial meniscus surgery	Histopathology: generally intact articular cartilage, OARSI score↓, Mankin score↓ Chondrogenic markers: Sox 9↑, Col2α1↑, aggrecan↑, HIF-1α↑ Signal pathway: Dvl1↑, β-catenin↑, GSK-3β↓ Inflammatory factors: IL-10↑, MMP-13↓ Pain-related indices: WBI↑, PWT↑	
Tang [70]	In vitro	Icariin	0.1, 1 and 10 μM	Oxygen‐glucose deprivation/reoxygenation‐induced rabbit BMSCs (icariin pretreatment)	BMSCs proliferation↑ Matrix formation: GAG↑ Chondrogenic markers: BMP2↑, aggrecan↑, Col2α1↑, actin cytoskeleton↑	Accelerating cartilage defect repair by promoting BMSC chondrogenic differentiation under the oxygen–glucose deprivation conditions
	In vivo	Icariin + BMSCs	10 μM	A cartilage defect on the rabbit trochlear surface of the femur	Histopathology: ICRS score↑, production of smooth surface and integrated cartilage structure, increase of chondrocytes and extracellular matrix Matrix formation: Col2α1↑	
Wang [71]	In vitro	Icaritin + GDF-5	5 μM	BMSCs in SD rats	Chondrogenic markers: aggrecan↑, Col2↑, Sox9↑ Signal pathway: DVL1↑, β-catenin↑, Gsk3β↓	Promoting BMSC chondrogenic differentiation through Wnt/β-catenin signaling pathway
Wang [72]	In vitro	Icariin + TGF-β3	1 µM	Rat BMSCs	Chondrogenic markers: Sox9↑, Col2↑, aggrecan↑ Fibrocartilage markers: Col1α1 (no increase) Hypertrophy markers: ALP (no increase)	Promoting chondrogenic differentiation with no effects on hypertrophy
Wang [73]	In vitro	Icariin + TGF-β3	1 µM	Rat BMSCs/self-assembling peptide nanofiber hydrogel scaffold	Chondrogenic markers: Sox9↑, Col2α1↑, Col2↑ Increase of chondrocyte-like rounded morphology Hypertrophy markers: ColX (no increase), ALP (no increase), Col1α1↓	Promoting BMSC stable chondrogenic differentiation in self-assembled peptide nanofiber hydrogel scaffolds
Luo [74]	In vivo	Icariin	25 mg/kg	OA model established by the transection of anterior cruciate ligament in C57/6J mice	Histopathology: subchondral bone formation↓, cartilage thickness↑, OASRI score↓ Matrix formation: GAG↑, Sox9↑, Col2α1↑ Hypertrophy markers: ColX↓, MMP13↓	Promoting chondrocyte differentiation and inhibiting chondrocyte hypertrophy by the down-regulation of IHH-related genes and the upregulation of PTHrP
	In vitro	Icariin	1 µM	Chondrocytes in C57/6J mouse OA model	Matrix formation: proteoglycan↑, Sox9↑, aggrecan↑ Bone formation markers: Runx2↓ Hypertrophy markers: MMP13↓, ColX↓ Signal pathway: PTHrP↑, IHh and its regulated genes (IHh↓, CyclinD1↓, Gli1↓, Ptch1↓)	
Bahrami [75]	In vitro	Icariin + TGF-β3	10 µM	Human AMSCs- fibrin constructions	Chondrogenic markers: Sox9↑, Col2↑, aggrecan↑ Fibrocartilage markers: Col1↓ Hypertrophy markers: ColX↓	Promoting AMSC chondrogenic differentiation and reducing hypertrophy effects
Yu [76]	In vivo	Icariin + AMSCs	6 × 10^4^ mg/L	Rabbit OA model established by the modified Hulth method	Histopathology: smooth and intact cartilage surface, Mankin score↓ Cell apoptosis: apoptosis rate of chondrocytes↓ Inflammatory factors: NO↓, IL-1↓, TNF-α↓ Signal pathway: TLR4↓, MyD88↓, TRAF6↓, p65↓	AMSCs with icariin pre-intervention can treat OA by inhibiting TLR4/NF-κB signaling pathway

#### Anti-inflammation and anti-OS

At present, more and more studies have shown that low-grade inflammation is closely related to OA [49]. Chondrocytes can not only respond to inflammatory factors in the joint tissue, but also produce proinflammatory factors themselves to further promote the release of degrading enzymes [49]. Tumor Necrosis Factor-α (TNF-α) and Interleukin- (IL-)1 are two crucial inflammatory cytokines in OA, which can promote the production of protease in chondrocytes [58, 77, 78]. Besides, they can also induce the synthesis of nitric oxide (NO) mediated by inducible nitric oxide synthase (iNOS), and prostaglandin mediated by COX-2 [58, 77, 78]. NO is another important player in OA and has several roles [77]. For example, NO can derive a series of highly oxidizing free radicals by interacting with compounds, such as reactive oxygen species (ROS), causing cell damage and apoptosis [79, 80].

Xianling Gubao capsules, made of five traditional Chinese herbs represented by *Epimedium*, have been widely used to treat osteoporosis and OA in China [81]. Ziadlou et al. selected 34 representative components from Xianling Gubao capsules [53]. Among the selected *Epimedium* components, OA chondrocytes 3D pellet culture with adding Epimedin C (25 µM) produced the most matrix (GAG/DNA) after 2 weeks. Further studies showed that 25 µM of Epimedin C can down-regulate COX-2 levels, reduce matrix degradation and promote matrix formation in human OA chondrocytes treated with IL-1β/TNF-α [53]. NF-κB and mitogen-activated protein kinase (MAPK) signaling pathways are key pathways involved in OA inflammation, especially the NF-κB pathway [57, 58, 60]. Hypoxia-inducible factor-2alpha (HIF-2α) is the downstream molecule of NF-κB, which directly induces the expression of MMP9, MMP13, ADAMTS-4, iNOS and prostaglandin-endoperoxide synthase-2 [82, 83]. Based on in vitro and in vivo experiments, the study of Wang et al. showed that icariin can down-regulate NF-κB/HIF-2α signaling pathway to relieve inflammation and protect chondrocytes [57]. Hyperoside is another flavonoid glycoside isolated from *Epimedium*. Sun et al. found that hyperoside at the concentration of 10, 20 and 40 μM can reduce iNOS and COX-2 expressions, decrease the production of ADAMTS5, MMP3 and MMP13, and enhance the expression of cartilage matrix formation factors in IL-1β-stimulated C57BL/6 mouse chondrocytes for 24 h [58]. The nuclear factor erythroid 2-related factor 2 (Nrf2) is a vital antioxidant factor in the cytoplasm and regulates the activity of various antioxidant enzymes, including superoxide dismutase (SOD), heme oxygenase-1 (HO-1), and NAD(P)H quinine oxidoreductase-1 (NQO-1) by binding to the antioxidant reaction elements in the nucleus [59]. Furthermore, by adding phosphatidylinositol 3 kinase (PI3K)/protein kinase B (AKT), extracellular signal-regulated kinase (ERK) and Nrf2 inhibitors, Sun et al. demonstrated that hyperoside can reduce inflammatory response and matrix loss in OA mainly by inhibiting PI3K/AKT/NF-κB and MAPK signaling pathways, and enhancing Nrf2/HO-1 signaling pathway [58]. In addition, they found that high ROS expression was associated with chondrocyte apoptosis, and hyperoside can reduce ROS production and chondrocyte apoptosis by up-regulating Nrf2 [58]. Zuo et al. pretreated human chondrocytes with icariin (10^–3^ μM) for 2 h and then stimulated with IL-1β [59]. Compared with direct IL-1β stimulation, they found that matrix degradation decreased in icariin pretreatment group. At the same time, ROS expression decreased, and Nrf2 and downstream antioxidant enzyme expression increased. Furthermore, they transfected human chondrocytes with Nrf2 siRNA, and they found the above phenomena and effects were eliminated. Therefore, they concluded that the regulation of Nrf2 is of great significance for the treatment of OA from the perspective of anti-inflammation and anti-OS [59].

Pain is still a thorny problem in OA, and there is no effective treatment yet [84]. Inflammation plays a vital role in causing pain [84]. Relevant studies have found that neuroregulation involved in regulating OA inflammation and pain [85]. *Epimedium* and its active ingredients can effectively inhibit OA inflammation, but whether they involve in the neuroregulation remains unclear. In the study of Li et al., they first confirmed the therapeutic effects of icariin on knee OA through in vivo and in vitro experiments, and exhibited the therapeutic effects are related to the inhibition of Toll-like receptor 4 (TLR4)/myeloid differentiation primary-response gene 88 (MyD88)/NF-κB inflammatory signaling pathway [61]. Second, based on functional magnetic resonance imaging and virus retrograde tracing techniques, they found that icariin can involve in the neural regulation by regulating the hypothalamic-mediated neuromodulation pathway and the endocannabinoid-related pathway. Further, by tandem mass tag-based quantitative proteomics and bioinformatics analyses, they verified levels of pain-related genes, such as Inα, Rtn4, changed after icariin treatment. Therefore, they concluded that icariin can participate in pain-related neural regulation of OA [61].

#### Inhibiting chondrocyte apoptosis, pyroptosis, and improving autophagy

The relationship between inflammation, OS, and apoptosis, pyroptosis and autophagy is very close. Apoptosis is programmed cell death to prevent excessive inflammation and damage to tissues. In OA, many factors such as inflammation, OS and autophagy state are related to chondrocyte apoptosis [86]. *Epimedium* and its active ingredients have been shown to inhibit chondrocyte apoptosis through various mechanisms.

The study of Mi et al. found that compared with the normal control group, the expressions of inflammatory cytokines (IL-1, IL-6, and IL-12), OS markers (NO, ROS), and apoptosis-related proteins (Bax, caspase-3, caspase-9) increased in TNF-α-treated SD rat chondrocytes. At the same time, the expression of autophagy markers (Atg5, Atg7, LC3-II) decreased [62]. However, the addition of icariin can partially block the above effects and is related to the inhibition of NF-κB pathway [62]. The non-coding RNAs (ncRNAs) is a kind of functional RNA that is transcribed from DNA but not translates into proteins [87]. More and more researches have shown that ncRNAs maintain the functions and homeostasis of cartilage, and also involve in the pathogenesis of OA [88]. The long non-coding RNAs (lncRNAs) refers to ncRNAs with a length of more than 200 nucleotides, which accounts for a considerable proportion of ncRNAs [87]. Wang et al. found that icariin can induce the upregulation of CYTOR, an IncRNA, to inhibit apoptosis of chondrocytes, and verified the findings by constructing CYTOR overexpression plasmids and CYTOR short hairpin RNA [63]. Zhou et al. studied the extraction method of *Epimedium* [64]. By using ethanol solution as extraction solvent and transfer rates of main flavonoids such as epimedins A–C, icariin and baohuoside I as evaluation index, they determined the optimal conditions for the ethanol extraction process of *Epimedium*, and demonstrated that the ethanol extract of *Epimedium* can reduce the apoptosis of chondrocytes induced by IL-1β [64]. Unlike apoptosis, the pyroptosis process is pro-inflammatory [89]. The typical pyroptosis pathway is mediated by caspase-1, in which nucleotide-binding oligomerization domain-like receptor family pyrin domain-containing protein 3 (NLRP3) inflammasome plays a key role [89]. Icariin has been shown to inhibit chondrocyte pyroptosis by inhibiting the expression of NLRP3 inflammasome and downstream factors, such as caspase-1 and GSDMD [56].

Autophagy, as a self-protection mechanism of cells, can use autophagosome and lysosome to remove damaged organelles and enhance the adaptabilities and survivals of cells [90]. Due to the unique structural characteristics of chondrocytes with absence of blood vessels, and lack of oxygen and nutrients, autophagy is particularly important in maintaining the structures and functions of chondrocytes [91, 92]. Targeting autophagy therapy is one of the hot topics in the treatment of OA [92]. Mammalian target of rapamycin (mTOR) is a negative regulator of autophagy, regulated by upstream PI3K/AKT and AMP-activated protein kinase (AMPK) signals [93]. In chondrocytes of OA rats established by the modified Hulth method, the activation of PI3K/AKT/mTOR pathway and increased chondrocytes apoptosis rate were observed [65]. After treatment with mTOR inhibitor, rapamycin, and different concentrations of icariin, the expression of PI3K/AKT/mTOR pathway-related factors decreased. However, the group of adding autophagy inhibitor, 3-methyladenine, showed significant chondrocytes apoptosis. The in vivo experiment results also exhibited that rapamycin and icariin can promote the expression of autophagy and improve the pathological status of OA articular cartilage [65].

Endoplasmic reticulum (ER) stress refers to the cellular state of protein misfolding and unfolded protein accumulation in ER caused by pathological conditions such as hypoxia, nutritional deficiency and continuous OS [94]. ER stress can activate autophagy to degrade misfolded proteins or damaged ER, and promote new ER generation, in order to maintain cell homeostasis [94, 95]. However, ER stress and long-term activation of autophagy tend to induce cell death [96]. Bone marrow-derived mesenchymal stem cells (BMSCs) have the ability to differentiate into chondrocytes after induction, which is one of the potential treatments for OA and cartilage defects [70]. However, in the OA environments with hypoxia and glucose deprivation, BMSCs will undergo ER stress, resulting in increased apoptosis, which greatly hinders the ability and efficiency of chondrogenic differentiation [67]. Icariin has been shown to reduce the expression of ER stress-related proteins, such as binding immunoglobulin protein (BIP), activating transcription factor 4 (ATF4) and XBP-1s (the active form of X-box-binding protein 1) in BMSCs through the inhibition of MAPK signaling pathway, thereby reducing the production of apoptosis proteins [67]. Besides, the expression of autophagy markers is also reduced, indicating that autophagy is also weakened [67]. In addition to enhancing the tolerance of cells to hypoxia and glucose deprivation by improving ER stress, icariin can also maintain the viability of chondrocytes in the hypoxia environment by increasing the expression of hypoxia-inducible factor-1α (HIF-1α), to promote glucose uptake and anaerobic glycolysis [68].

#### Promoting chondrogenic differentiation of MSCs

With the development of regenerative medicine, stem cell and cytokine therapy has gradually become a new choice for OA treatment [69]. Cartilage tissue engineering utilizes the potential of MSCs to differentiate into chondrocytes. These MSCs include BMSCs, adipose-derived MSCs (AMSCs), synovial membrane-derived MSCs (SM-MSCs), Umbilical cord blood-derived MSCs (UCB-MSCs), and induced pluripotent stem cells-derived MSCs (IPSC-MSCs) [97, 98]. *Epimedium* and its active ingredients play an important role in inducing chondrogenic differentiation of these MSCs, and reducing hypertrophy.

BMSCs are ideal, most widely studied source of autologous cell therapy for promoting cartilage repair [99]. Zhu et al. found in vitro that hydrogels loaded with different concentrations of icariin can promote BMSCs chondrogenic differentiation in varying degrees, and selected icariin at a concentration of 147.8 μM as the optimal concentration for in vivo experiments [69]. Then, the OA model was established by destabilizing medial meniscus surgery in SD rats, and BMSCs-loaded icariin hydrogel was injected into the articular cavity. Compared with the PBS group, the BMSCs group, and the icariin hydrogel group, the cartilage morphology of the BMSCs-loaded icariin hydrogel group was complete, and the expression of chondrogenic markers, such as Sox 9, Col2α1 and aggrecan increased. At the same time, the matrix metalloproteinase MMP13 decreased. Moreover, the factors related to wingless-related integration site (Wnt)/β-catenin pathway changed. These results demonstrated that icariin-loaded hydrogel can promote the chondrogenic differentiation of BMSCs and was related to the activation of Wnt/β-catenin pathway [69]. In addition, icariin can increase the tolerance of BMSCs to ischemia and hypoxia, increasing the long-term benefit of OA [70]. In addition to icariin, icaritin has also shown to promote BMSCs chondrogenic differentiation via the wnt/β-catenin pathway [71]. Hypertrophy is a common side effect of using growth factors such as transforming growth factor- (TGF-) β to induce MSCs differentiation [72]. X-type collagen (ColX), MMP13, osteocalcin, Indian hedgehog (IHh), and alkaline phosphatase are verified markers of hypertrophic chondrocytes [100]. Wang et al. found that after adding icariin, the ability of TGF-β to promote BMSCs chondrogenic differentiation enhanced, but did not increase hypertrophy reaction and fibrochondrogenesis [72]. There is a negative feedback regulation between parathyroid hormone-related protein (PHrP) and IHh [74]. In OA, chondrocytes acquire hypertrophy phenotype, and the expression of IHh increases [74, 100]. The increased IHh will up-regulate PHrP, and in turn inhibit IHh expression to maintain cartilage homeostasis [74, 100]. In the study of Luo et al., icariin was shown that can promote chondrocyte differentiation, and up-regulate PTHrP and down-regulate IHh to inhibit chondrocyte hypertrophy [74].

AMSCs are easier to obtain and may have better chondrogenic differentiation potential than BMSCs [75]. Bahrami et al. cultured TGF-β and/or icariin with human subcutaneous AMSC-fibrin constructors, and found that the icariin + TGF-β group can significantly increase the chondrogenic differentiation ability of the constructors, and the expression of hypertrophic markers (ColX) and fibro-chondrogenic markers (type I collagen) decreased compared with the TGF-β group [75]. Yu et al. injected 1 mL of autologous subcutaneous AMSC suspension, which was treated with 60 g/L icariin solution for 3 days, into the joint cavity of rabbit OA [76]. They found that compared with injecting saline, and injecting subcutaneous AMSCs without icariin pretreatment, the expression of NO, IL-1 and TNF-α in joint fluid decreased significantly after 14 days. Besides, the cartilage surface was intact, and the Mankin score of cartilage injury decreased. Moreover, they found that the expression of factors related to TLR4/NF-κB pathway decreased. Therefore, they concluded that icariin combined with AMSCs can treat OA [76].

SM and cartilage originate from a common cell pool during joint development, which means that SM-MSCs are more prone to chondrogenesis than BMSCs and AMSCs [101]. Zare et al. injected SM-MSCs and AMSCs into the articular cavity of OA rats, and found that they both improved radiological and pathological grading of articular cartilage [102]. By comparison, SM-MSCs showed better results [102]. UCB-MSCs are more accessible to obtain through painless extraction procedures [97]. Li et al. conducted a meta-analysis of clinical studies on UCB-MSCs in cartilage defects and OA treatment, and confirmed the efficacy and quality of UCB-MSCs treatment [103]. IPSCs refer to reprogramming terminally differentiated adult cells into PSCs by introducing specific transcription factors [98]. It is reported that IPSCs showed similar properties with embryonic stem cells in self-renewal and differentiation capacity [98]. Diederichs et al. reprogrammed human BMSCs to IPSCs and then derived MSCs [104]. They found that these stem cells had the morphology and surface markers of MSCs, but exhibited high heterogeneity compared with the originating parental BMSCs. In addition, they were less responsive to traditional BMSCs differentiation protocols. Therefore, the authors believe that the MSCs generated from IPSCs are a unique population of cells with mesenchymal characteristics [104]. Although the effects on IPSC-MSCs are still being determined, they are an attractive and promising source of cells considering their almost inexhaustible nature.

Although the above cell therapies have their own advantages, it is challenging to maintain stable heredity and phenotype [105]. Therefore, many medical scientists have turned their attention to exosomes (Exos) [105]. Exos is a kind of extracellular vesicle secreted by cells, containing proteins, messenger RNA, microRNA and other substances, which is the carriers of cell communication [106]. MSCs can indirectly act on target cells by secreting Exos, which is important in disease treatment [105]. Zhu et al. injected IPSC-MSC-Exos and SM-MSC-Exos into collagenase-induced OA mice, and found that both Exos promoted chondrocyte proliferation and migration. In contrast, IPSC-MSC-Exos worked better [101].

Many studies have reported the advantages and disadvantages of MSCs derived from different sources in chondrogenic differentiation. However, there are few studies on the role of *Epimedium* and its active ingredients in promoting chondrogenic differentiation of synovial membrane-derived, umbilical cord blood-derived, and induced pluripotent stem cells-derived MSCs. Besides, there are few studies about OA treatment with *Epimedium* by regulating Exos. In the future, we expect more studies will focus on the above three stem cells in chondrogenic differentiation and the intervening role of *Epimedium*. Also, whether Exos mechanism participates in the *Epimedium* treating OA is worth studying.

### Improving synovial inflammation

SM is a connective tissue membrane attached to the edge of articular cartilage and adhered to the joint capsule, which can be divided into the inner synovial layer and the sub-synovial layer [107, 108]. The inner synovial layer is dominated by cells, mainly composed of fibroblasts, macrophages and MSCs [107, 108]. The sub-synovial layer is dominated by blood vessels, lymph and nerves [107, 108]. SM can regulate the composition of joint fluid and provide nutrients to cartilage [108].

Synovitis is the main pathological process of SM, and is closely related to OA. In a prospective epidemiological study, synovitis was present in a majority of OA patients and was associated with pain and dysfunction in patients [109, 110]. It has been found that synovitis may be an early change of OA, and crosstalk between SM and cartilage plays an important role in OA progression [111]. Tissue debris from meniscus injury, anterior fork ligament injury, and cartilage degradation products all have the potential to trigger synovitis [112, 113]. Then, cytokines and proteases released by SM will cause cartilage to release more cytokines and proteases, resulting in progressive cartilage loss and persistent inflammation of SM, forming a vicious cycle [112, 113]. By summarizing the studies of *Epimedium* and its active ingredients in the treatment of OA, as detailed in Table 3, we found that they can treat synovial inflammation in OA through various mechanisms.

**Table 3:** Studies of *Epimedium* and its active ingredients in improving OA synovial inflammation

References	Assay type	Component of *Epimedium*	Concentration/dosage	Cells/model	Effects	Mechanisms
Pan [114]	In vitro	Icariin	0.1, 0.5 and 1 μM	Fibroblast-like synovial cells in OA patients	Proliferation and migration of OA-fibroblast-like synovial cells↓ Matrix degradation: IL-1β↓, MMP14↓ ER stress-related protein: BIP↓	Regulating cellular functions and gene expression of Fibroblast-like synovial cells in OA patients
Jin [115]	In vivo	Total flavonoids of *Epimedium*	150, 300 and 600 mg/kg	OA rat model established by the transection of anterior cruciate ligament	Pain related indices: PWT↑ Knee joint diameter↓ Inflammatory factors: IL-6↓, IL-1β↓, TNF-α↓ Histopathology: inflammatory cell infiltration in synovial tissue↓, Synovial hyperplasia↓ Signal pathway: p-IκB-α/IκB-α↓, p-p65/p65↓	Relieving synovial inflammation by inhibiting NF-κB signal pathway in rat OA
Hu [116]	In vitro	Quercetin	2, 4 and 8 μM	IL-1β treated chondrocytes in the knee joint of SD rats	Chondrocyte proliferation↑ Matrix formation: aggrecan↑, Col2α↑, GAG↑ Inflammatory factors: PGE2↓, NO↓, iNOS↓, COX-2↓ Matrix degradation: MMP13↓, ADAMTS-4↓ Signal pathway: IκBα↑, p-AKT/AKT↓, p-P65/P65↓, nuclear P65↓	1. Anti-inflammation by inhibiting the Akt/NF-κB signaling pathway 2. Promoting the polarization of synovial macrophages to M2 macrophages, to create a microenvironment for chondrogenesis
	In vitro	Quercetin	8 μM	RAW 264.7 cells	M2 macrophage-related genes: Arg1↑, MR↑, Ym1↑ Pro-chondrogenic cytokines: TGF-β1↑, TGF-β2↑, TGF-β3↑, IGF-1↑, IGF-2↑ Signal pathway: pSTAT6↑, p-AKT↑	
	In vivo	Quercetin	8 μM, 0.1 mL	OA rat model established by removing the medial meniscus and transecting the anterior meniscotibial ligament	Histopathology: structural integrity of cartilage↑, OARSI score↓ Matrix formation: GAG↑, aggrecan↑, Col2α↑ Matrix degradation: MMP13↓ Cell apoptosis: chondrocyte apoptosis↓ M2 macrophage marker (synovial tissues): MR↑ Pro-chondrogenic cytokines (synovial fluid): TGF-β1↑, TGF-β2↑	
Luo [117]	In vitro	Icariin	2, 5 and 10 µM	LPS-induced human synovial cells (HUM-CELL-0060)	Synovial cell mortality↓, cell viability↑ Ferroptosis-related factors: iron content↓, MDA↓, TFR1↓, NCOA4↓ Antioxidant-related factors: GPX4↑, Nrf2↑ Signal pathway: SLC7A11↑, GPX4↑, SLC3A2L↑	Treating synovitis by activating the Xc−/GPX4 axis to inhibit ferroptosis

Fibroblasts are the dominant cell population in the SM, and their excessive proliferation is related to the release of inflammatory factors and the secretion of matrix degrading enzymes [118]. Pan et al. treated human fibroblast-like synovial cells in OA patients with different concentrations of icariin, and found that icariin can inhibit the proliferation and migration of fibroblast-like synovial cells in a dose-dependent manner [114]. In addition, the expression of IL-1, MMP14 and BIP, which are related to cartilage matrix degradation and ER stress also decreased [114]. Jin et al. established OA models by transecting the anterior cruciate ligament of the right knee joint in SD rats, and then treated with total flavonoids of *Epimedium* by gavage for 4 weeks [115]. They found that compared with the model control group, the total flavonoids of *Epimedium* group can relieve knee swelling, and increase pain threshold in OA rats. Besides, the synovial inflammatory cell infiltration and hyperplasia reduced, and the serum levels of IL-6, IL-1β and TNF-α decreased. Jin et al. also proved that the above effects were related to the inhibition of NF-κB signaling pathway [115]. Synovial macrophages, another important synovial cells, maintain the dynamic balance of anti-inflammation and pro-inflammation in synovial fluid through MI and M2 polarization states, with M1 promoting inflammation, and M2 inhibiting inflammation [119]. Quercetin is a flavonoid compound found in various herbs, including *Epimedium*. In vitro, quercetin at the concentrations of 2, 4, and 8 μM have been shown to inhibit the expression of inflammatory cytokines, the production of matrix-degrading enzyme, and chondrocyte apoptosis in a dose-dependent manner by inhibiting the AKT/NF-κB signaling pathway [116]. In addition, 8 μM quercetin can polarize RAW 264.7 cells into M2 macrophages, and release factors that promote cartilage growth, such as TGF-β1, TGF-β2, TGF-β3, insulin-like growth factor- (IGF-) 1, and IGF2, providing a microenvironment for chondrogenesis. In vivo experiments also confirmed the results obtained in vitro [116].

Ferroptosis is iron-dependent cell death associated with lipid peroxidation [120]. That is to say, iron accumulation and lipid peroxidation are crucial factors of ferroptosis [120]. Through the Fenton reaction, Fe^2+^ triggers lipid peroxidations to produce lipid hydroperoxides [121]. Malondialdehyde (MDA) is the main secondary metabolite of lipid hydroperoxides [121]. Glutathione (GSH) is a crucial antioxidant in the body, and cystine is the raw material of its synthesis [120, 121]. The cystine/glutamate antiporter system Xc− on the cell membrane can transport cystine into the cell, in which SLC7A11 as a system Xc− subunit, is highly specific for cystine/glutamate, thus facilitating the GSH formation [120, 121]. GSH peroxidase 4 (GPX4) is a GSH-dependent enzyme that ultimately mediates the reduction of lipid hydroperoxide or free hydrogen peroxide [120]. Yu et al. discovered that iron accumulation and decreased expression of GSH, GPX4, SLC7A11 and other factors related to antioxidant in human OA cartilage through the transcriptome RNA sequencing technology, indicating that ferroptosis is closely related to OA [122]. Synovitis is an important pathological process of OA. Luo et al. studied whether icariin can protect synovial cells by inhibiting ferroptosis to prevent OA development [117]. They found that icariin treatment of LPS-induced human synovial cells decreased the iron content, MDA and other factors related to ferroptosis, while increased the antioxidant-related factors such as GPX4, Nrf2 and SLC7A11. Besides, icariin counteracted the increased iron content and the imbalance between oxidate and antioxidant systems caused by GPX4 inhibitor-RSL3. Therefore, they concluded that icariin can protect synovial cells by inhibiting ferroptosis through Xc−/GPX4 axis [117].

### Regulating subchondral bone remodeling

Cartilage and subchondral bone are not two separate joint tissues [123]. They are closely related in structure and function [123]. The calcified cartilaginous layer of cartilage is directly connected to the subchondral bone plate in a “comb tooth” structure [124]. The subchondral bone plate is a cortical lamellar bone structure that can withstand the stress directly from cartilage [125]. The subchondral cancellous bone (trabeculae) is distributed and arranged in the same direction as the stress and tension on the subchondral bone, helping to absorb joint stress and transfer them to the surroundings evenly [124, 125]. Guévremont et al. detected hepatocyte growth factor in OA cartilage [126]. However, it is not produced by chondrocytes, but by osteoblasts from subchondral bone plate, which indicates that there is a biochemical connection between cartilage and subchondral bone, and the calcified cartilage is effective on transporting such factors [126]. In conclusion, cartilage and subchondral bone, as a functional unit, are interconnected. Subchondral bone targeting therapy provides a new therapeutic idea for OA [127].

Osteoclasts dismantle and carry away old bone, and osteoblasts rebuild new bone. This process is called bone remodeling. In the early stage of OA, subchondral bone showed increased bone resorption and rapid bone remodeling, leading to structural changes such as decreased subchondral bone plate thickness, and increased porosity [128–131]. However, in the late stage of OA, subchondral bone showed decreased bone resorption and remodeling, and the bone formation relatively increased, with the image feature exhibiting subchondral osteosclerosis [128–131].

*Epimedium* is a common Chinese herb for the treatment of osteoporosis, and there are many studies on its effects on osteoporosis. It can promote osteogenic differentiation of BMSCs, promote bone formation, or inhibit bone resorption through various mechanisms to treat osteoporosis [132–134]. However, in the environment of OA, the effects of *Epimedium* and its ingredients on bone remodeling are limited. Gao et al. used the transection of anterior cruciate ligament method to establish OA mice models [135]. After 8 weeks, they found that the indicators of bone remodeling (CTX, osteocalcin) decreased. Histopathology showed the late OA manifestations, such as extensive exposure of subchondral bone, cartilage loss, thickening of subchondral cortical bone plate, and reduction of bone trabecula numbers. After treatment with icariin, bone remodeling of OA mice was regulated, and morphological structure of cartilage and subchondral bone was improved, especially early administrating icariin [135]. TGF-β plays a vital role in bone remodeling, which is secreted by osteoblasts and deposited in bone matrix [136]. Osteoclasts can activate it during bone resorption [136, 137]. Subsequently, TGF-β specifically induces MSCs recruitment to bone resorption sites and promotes their proliferation through signal transduction protein SMAD family, thus contributing to bone formation [136, 137]. Elevated TGF-β1 was observed in both animal and human OA, and has been shown to be associated with abnormal bone formation, OA severity, angiogenesis, and mineralization reduction [138, 139]. Xu et al. established OA rat models by modified Hulth method, and then administered icariin intragastrically for 12 weeks [140]. They found that the disorder of bone metabolism in OA rats was regulated, with 1,25-(OH)_2_D_3_, CTX, and osteocalcin increased, and the morphological structures of cartilage and subchondral bone improved. In addition, they found that factors related to the TGF-β signaling pathway decreased after treatment, suggesting that the therapeutic effects of icariin may be related to the inhibition of the TGF-β signaling pathway [140].

## Toxicity

Unlike *Tripterygium wilfordii* Hook.f., its toxicity is widely known in TCM [141]. In recent years, with the increased reports of adverse reactions to *Epimedium* and its proprietary Chinese medicines, *Epimedium* is considered as a potential toxic drug. By searching and summarizing the toxicity of *Epimedium* and its main components, we found that the toxicity of *Epimedium* is mainly related to the flavonoids, and hepatotoxicity was more reported [142–144]. Cao combined with network toxicology and cellular experiments to target and verify that icaritin is the material basis of hepatotoxicity [145]. Further, through metabolomics technology, she considered that the mechanism might be related to GSH metabolism. In addition, the study of Cao also found that the combination with *Ligustrum lucidum* W.T.Aiton, another common kidney tonic herb, can inhibit the enrichment of the main flavonoids of *Epimedium* in the body, suggesting that the combination of the two is of great importance [145]. In addition, *Morinda officinalis* F.C. How has also been reported to have a detoxification effect on *Epimedium* [146]. Yang et al. conducted a study on the content of heavy metals in *Epimedium* [147]. They found that the content of heavy metals varied greatly among different species of *Epimedium*, with copper and lead being the main heavy metals, and the levels of copper and lead were higher in wild *Epimedium* than in cultivated *Epimedium*. In addition, they found that some samples sold in the Chinese market had excessive heavy metal content. Therefore, they suggested that toxic heavy metal contamination is also an issue that needs attention in the application of *Epimedium* [147]. Xianling Gubao Capsule is a proprietary Chinese medicine that has been widely used in the treatment of bone diseases such as fractures and osteoporosis in recent years, and is also the proprietary Chinese medicine with the most reported adverse reactions to *Epimedium* preparations in recent years [148]. Peng analyzed 2796 reports of adverse drug reactions/adverse events (ADRs/AEs) after using Xianling gubao capsule/tablet from the National Adverse Drug Reaction Monitoring Center from 2004 to mid-2006, and found that ADRs/AEs mainly manifested as nausea, rash, itching, gastrointestinal disorders, and dizziness, and severe ADRs/AEs mainly manifested as abnormal liver function, accounting for 44.12% of all severe ADRs/AEs manifestations [149]. Based on the above, it is suggested that liver functions of patients should be paid close attention when the Xianling gubao capsule is used in clinical practice.

However, several toxicity tests have shown no acute, subchronic toxicity or genotoxicity of *Epimedium* extraction or its major ingredients [150–152]. The clinical manifestations, blood physiological and biochemical parameters, histopathology, and cell proliferation capacity of the test animals were not significantly different from those of the control animals. The differences in results may be related to the species, extraction method, processing method, dosage, duration, and compatibility of *Epimedium* [148]. Gao et al. reviewed the literature on *Epimedium* toxicity and proposed a drug-specific response theory of *Epimedium* [153]. In other words, there is no obvious direct toxicity of this drug, and the occurrence of toxicity is mainly related to the patient’s metabolism or immune idiosyncrasy. If the body is under immune stress, it is more likely to trigger hepatotoxicity.

In summary, *Epimedium* is relatively safe in clinical use, but cautions should be exercised to avoid taking large dosages or long periods. Besides, attentions should be paid to patient’s own immune and metabolic states when using *Epimedium*.

## Conclusions

OA is a complex disease, which has not been effectively conquered clinically. At present, there are many studies on OA therapeutic targets. TCM has the characteristics of multiple targets, and has great prospects in treating OA. *Epimedium* is one of the commonly used kidney tonic herbs, which can nourish the liver and kidney, strengthen the muscles and bones, dispel wind and dampness, and can effectively treat both the symptoms and the root cause of OA. Through searching and summarizing literature on the treatment of OA by *Epimedium* and its ingredients, we found that their treatments of OA are relatively comprehensive, involving various pathological processes and signaling pathways. We reviewed the therapeutic effects of *Epimedium* and its active ingredients on OA from three aspects: maintaining cartilage structures and functions, improving synovial inflammation, and regulating subchondral bone remodeling. However, we also found that there are some deficiencies in the treatment of OA with *Epimedium*. For example, the bioavailability of main flavonoids in *Epimedium* are not high, and the studies on OA treatment mainly focus on flavonoids, especially icariin, and there are few studies on other ingredients, such as *Epimedium* polysaccharides, and *Epimedium* lignans. Therefore, improving the bioavailability of flavonoids in *Epimedium* by chemical modification and other methods, and paying more attentions to other components that have the potential to treat OA, are the key to enhancing the application value of *Epimedium* in OA treatment. In addition, Exos have increasingly become the hot topic in the field of regenerative medicine, and play an important role in the progress and treatment of OA [154, 155]. Whether *Epimedium* and its ingredients can treat OA through Exos mechanism is also one of the future research directions.

## Data Availability

The data used to support the findings of this study are available from the corresponding author upon request.

## Abbreviations

OA: Osteoarthritis
TCM: Traditional Chinese Medicine
ROS: Reactive oxygen species
NLRP3: Nucleotide-binding oligomerization domain-like receptor family pyrin domain-containing protein 3
Col2: Type II collagen
GAG: Glycosaminoglycan
MMPs: Matrix metalloproteinases
ADAMTS: ADAM with thrombospondin-1 domains
OS: Oxidative stress
MSCs: Mesenchymal stem cells
TNF-α: Tumor Necrosis Factor-α
IL-: Interleukin-
NO: Nitric oxide
Inos: Inducible nitric oxide synthase
COX-2: Cyclooxygenase-2
NF-: Nuclear factor-
MAPK: Mitogen-activated protein kinase
HIF-2α: Factor-2alpha
Nrf2: Nuclear factor erythroid 2-related factor 2
SOD: Superoxide dismutase
HO-1: Heme oxygenase-1
NQO-1: NAD(P)H quinine oxidoreductase-1
PI3K: Phosphatidylinositol 3 kinase
AKT: Protein kinase B
ERK: Extracellular signal-regulated kinase
TLR4: Toll-like receptor 4
MyD88: Myeloid differentiation primary-response gene 88
ncRNAs: Non-coding RNAs
lncRNAs: Long non-coding RNAs
mTOR: Mammalian target of rapamycin
AMPK: AMP-activated protein kinase
ER: Endoplasmic reticulum
BMSCs: Bone marrow-derived mesenchymal stem cells
BIP: Binding immunoglobulin protein
ATF4: Activating transcription factor 4
HIF-1α: Hypoxia-inducible factor-1α
AMSCs: Adipose-derived MSCs
SM-MSCs: Synovial membrane-derived MSCs
UCB-MSCs: Umbilical cord blood-derived MSCs
IPSC-MSCs: Induced pluripotent stem cells-derived MSCs
Wnt: Wingless-related integration site
TGF-: Transforming growth factor-
ColX: X-type collagen
IHh: Indian hedgehog
PHrP: Parathyroid hormone-related protein
Exos: Exosomes
IGF-: Insulin-like growth factor-
MDA: Malondialdehyde
GSH: Glutathione
GPX4: GSH peroxidase 4
ADRs/AEs: Adverse drug reactions/adverse events
